# Clinical significance of procoagulant microparticles

**DOI:** 10.1186/s40560-014-0066-z

**Published:** 2015-01-07

**Authors:** Shosaku Nomura, Michiomi Shimizu

**Affiliations:** First Department of Internal Medicine, Kansai Medical University, 2-3-1 Shin-machi, Hirakata, Osaka 573-1191 Japan

**Keywords:** Microparticle, Procoagulant activity, Phospholipid, Thrombosis, Flow cytometry

## Abstract

Microparticles (MPs) are small membrane vesicles that are released from many different cell types by exocytic budding of the plasma membrane in response to cellular activation or apoptosis. MPs may also be involved in clinical diseases because they express phospholipids, which function as procoagulants. Although flow cytometry is the most widely used method for studying MPs, some novel assays, such as tissue factor-dependent procoagulant assay or the ELISA method, have been reported. However, the use of quantification of MP as a clinical tool is still controversial. Elevated platelet-derived MP, endothelial cell-derived MP, and monocyte-derived MP concentrations are documented in almost all thrombotic diseases occurring in venous and arterial beds. However, the significance of MPs in various clinical conditions remains controversial. An example of this controversy is that it is unknown if MPs found in peripheral blood vessels cause thrombosis or whether they are the result of thrombosis. Numerous studies have shown that not only the quantity, but also the cellular origin and composition of circulating MPs, are dependent on the type of disease, the disease state, and medical treatment. Additionally, many different functions have been attributed to MPs. Therefore, the number and type of clinical disorders associated with elevated MPs are currently increasing. However, MPs were initially thought to be small particles with procoagulant activity. Taken together, our review suggests that MPs may be a useful biomarker to identify thrombosis.

## Introduction

Microparticles (MPs) are small membrane vesicles that are released from many different cell types by exocytic budding of the plasma membrane in response to cellular activation or apoptosis [1-3]. MPs disseminate various bioactive effectors originating from the parent cells. Therefore, MPs can alter vascular function and may induce biological responses involved in vascular homeostasis [4]. Although most MPs in human blood originate from platelets, MPs are also released from leukocytes, erythrocytes, endothelial cells, smooth muscle cells (SMCs), and cancer cells [5-10]. MP concentrations are documented in almost all thrombotic diseases occurring in venous and arterial beds [11-14]. Elevated levels of MPs have also been found in a number of conditions associated with inflammation, cellular activation and dysfunction, angiogenesis, and transport [15-23]. In this review, we address the function of MPs and some of the clinical findings that suggest important roles for procoagulant MPs.

## Review

### Composition and production of MPs

The standard platelet-derived MP (PDMP) measurement by flow cytometry was demonstrated by the International Society of Thrombosis and Haemostasis [24,25]. According to this committee, MPs can range in size from 0.1 to 1.0 μm. The membrane composition of MPs reflects the membranous elements of the cell of origin (Table 1). PDMPs contain molecules in addition to glycoproteins (GPs), such as platelet-activating factor, β-amyloid precursor protein, Ca^2+^-dependent protease calpain, arachidonic acid, and many phospholipids [26-31]. Phospholipids are particularly important because they are involved in the function of PDMPs. Furthermore, PDMPs serve as a finely tuned transcellular delivery system for the chemokine regulated on activation, normal T-cell expressed and secreted (RANTES) [32].

**Table 1:** **Origin and antigens of MPs**

**Origin**	**Antigen**
*Erythrocyte*	CD235a (glycophorin A)
*Platelet*	CD42a (GPIX)
	CD42b (GPIb)
	CD41 (GPIIb/IIIa, *α*_IIb_ *β*_3_)
	CD61 (GPIIIa)
	CD62P (P-selectin)
*Leukocyte*	
Neutrocyte	CD66b (CEACAM-1)
Monocyte	CD14 (endotoxin receptor)
Lymphocyte	CD4, CD8, CD20
*Endothelial cell*	CD31 (PECAM-8)
	CD51 (vitronectin receptor, *α*_v_ *β*_3_)
	CD54 (ICAM-1)
	CD62E (E-selectin)
	CD105 (endoglin)
	CD144 (VE-cadherin)
	CD146 (MelCAM)

MPs contain functional cytoadhesions, bioactive phospholipids, cytoplasmic components, and various antigens that are characteristic of the state of the cell from which they originated and also of the type of stimulus [33,34]. Some studies have analyzed the proteome of MPs and identified hundreds of proteins [35,36]. Proteins from MPs may be useful biomarkers for various disease processes [36].

**Figure 1:**
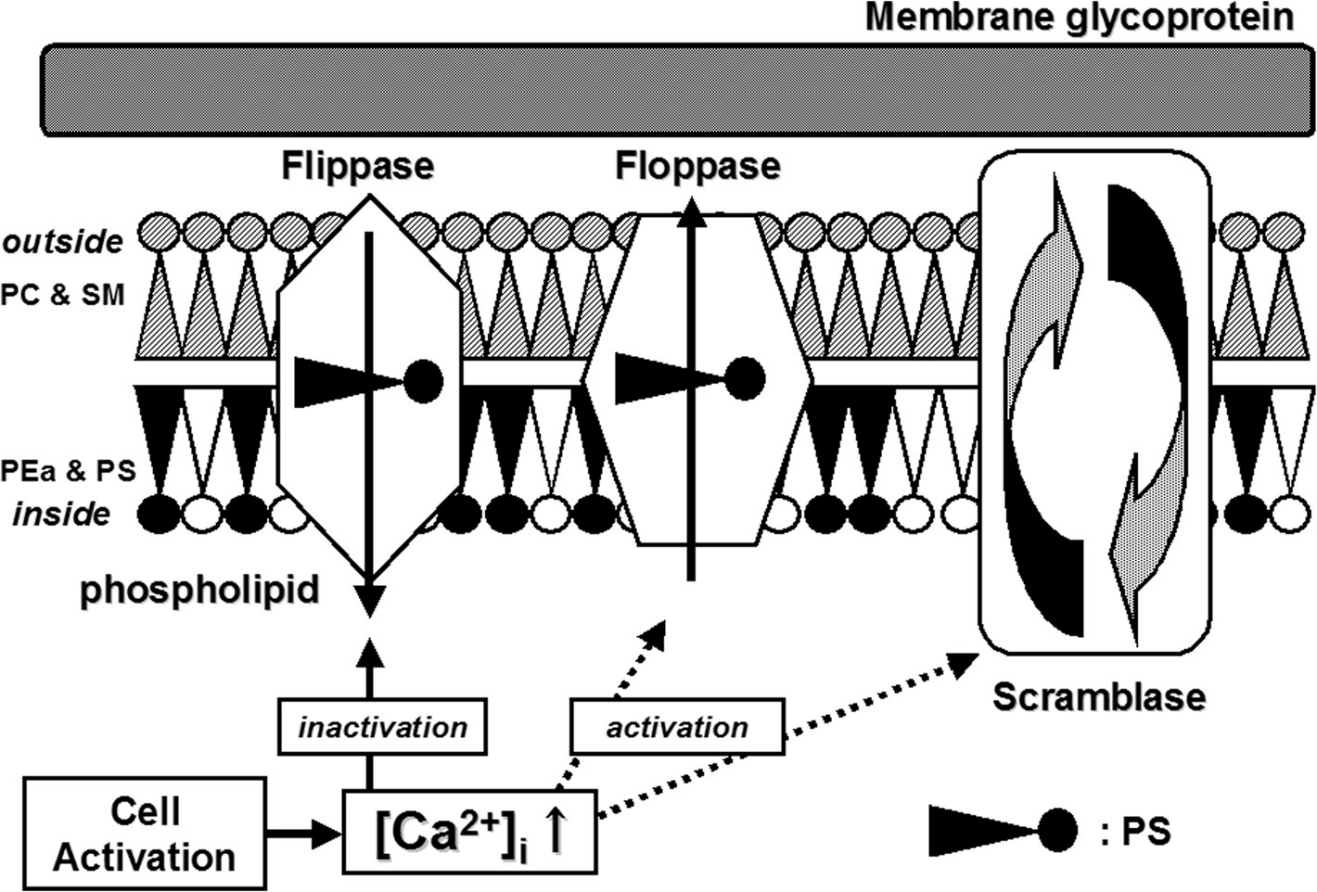
**Mechanisms participating in the regulation of transmembrane migration of phosphatidylserine (PS) in activated platelets, followed by PDMP shedding.** Phospholipid asymmetry is under the control of active flippase, while floppase and scramblase remain inactive. Following cellular activation, calcium is released from the endoplasmic reticulum, which can lead to the loss of phospholipid asymmetry and activation of calpain. PC, phosphatidylcholine; SM, sphingomyeline; PEa, phosphatidylethanolamine.

MPs are constitutively released from the surface of cells, but their formation can be upregulated by cellular activation or apoptosis [37,38]. After cellular activation or apoptosis is triggered, there is a rise in cytosolic calcium concentrations followed by cytoskeletal changes. Many studies have shown that calpain activation is important for PDMP generation [39,40]. Additionally, calpain appears to help limit phosphatidyl inositol phosphate (PIP)_2_ formation following platelet activation, and PIP_2_ content is an important determinant of PDMP formation [41,42]. However, some reports have suggested the existence of a distinct mechanism of calpain activation [43,44]. Plasma membranes of cells contain different types of phospholipids. Although uncharged phospholipids are mainly present in the outer leaflet of the membrane bilayer, the inner leaflet contains negatively charged aminophospholipids, such as phosphatidylserine (PS). During activation or apoptosis of the cell, there is a change in the membrane with alteration in the normal lipid bilayer, ‘flip-flopping’ the internal PS to the external surface. As a result, PS-exposing MPs may be released from cells (Figure 1) [45].

High shear stress can initiate platelet aggregation and shedding of procoagulant-containing PDMPs [46]. Chow et al. [47] suggested that thrombin that is formed in the vicinity of primary hemostatic plugs in areas of elevated shear stress plays a major role in the propagation of thrombi by potentiating shear-induced PDMP generation. Furthermore, platelet GPs and specific receptors can be involved in high shear stress-induced PDMP formation [46,48-50]. Miyazaki et al. [46] examined the mechanisms involved in PDMP production induced by high shear stress and showed that binding of von Willebrand factor to GPIb, influx of extracellular calcium, and activation of platelet calpain were required to generate PDMPs under conditions of high shear stress. In addition, Reininger et al. [50] recently reported that the GPIb receptor mediates adhesion to von Willebrand factor, and under hydrodynamic flow, stretching of the platelet membrane occurs, followed by separation of areas of tethered membranes and production of MPs. Shear stress is also involved in the mechanism of PDMP generation because it is a major determination of endothelial apoptosis [51,52].

MPs are similar to damage-associated molecular patterns (DAMPs). DAMPs are normally hidden within live cells and are released from dying or damaged cells [53]. The typical DAMPs are a high mobility group box 1 (HMGB1)[54]. HMGB1 is normally located in the nucleus where it acts as a DNA chaperon by regulating transcription [55]. However, the extracellular HMGB1 is a substance itself and works tissue injuriously for normal cell or organ [56]. In contrast, the role of MPs is a carrying system of tissue factor (TF), cell adhesion molecules, chemokines, and HMGB1 [56,57]. However, the differences between MPs and DAMPs are actually confused.

### Effective roles of MPs for coagulation

MPs were initially thought to be related to disease because they express phospholipids, which are procoagulants. These MPs support generation of thrombin and could be involved in diffuse intravascular coagulation occurring in disease states. However, such a coagulation system is activated not only in disease states, but also in healthy individuals. Berckmans et al. [58] reported that MPs circulate in healthy humans and support low-grade thrombin generation. Sinauridze et al. [59] reported that PDMPs have 50- to 100-fold higher specific procoagulant activity than activated platelets. Exposure of PS not only facilitates formation of coagulation complexes, but also promotes the ability of TF to initiate coagulation [60].

MPs support coagulation by factor (F)VII/TF-dependent and independent pathways [61]. During vascular damage, blood contacts extravascular TF, resulting in extrinsic coagulation activation and formation of fibrin. Indeed, TF can become active upon adhesion and fusion of MPs to activated platelets.

Several studies found that stimulation by tumor necrosis factor (TNF)-α, lipopolysaccharide, or oxidized low-density lipoprotein in cultured human umbilical vein endothelial cells results in an increase in the release of endothelial cell-derived MPs (EDMPs) expressing surface TF [7,62,63]. The addition of increasing concentrations of these EDMPs to a coagulation assay shortens the plasma clotting time. This effect is not observed in FVII-deficient plasma, indicating that procoagulant activity of EDMPs is FVII/TF dependent in this situation. Interestingly, a subset of EDMPs bearing von Willebrand factor appears to be able to induce platelet aggregation [64].

Monocyte-derived MPs (MDMPs) also contribute to the development of platelets and fibrin-rich thrombus at sites of vascular injury, through the recruitment of cells and accumulation of TF. MDMPs express the P-selectin glycoprotein ligand-1 and TF [65]. The binding of these MDMPs to P-selectin on activated endothelial cells on activated platelets within the developing thrombus may promote accumulation of TF and localized thrombin generation. TF-exposing monocytes may also release TF-exposing MDMPs [66]. Subsequently, activated platelets expose P-selectin and are capable of capturing TF-exposing MDMPs via P-selectin glycoprotein ligand-1. Consequently, MP-associated TF becomes rapidly deposited at the site of the developing thrombus. DelConde et al. [67] showed *in vitro* that fusion of membranes of TF-exposing MPs and activated platelets results in the transfer of TF in platelet membranes. This fusion results in co-localization of TF and coagulation factors, thereby promoting a more efficient initiation and propagation of coagulation.

At the MP surface, the presence of proteins inhibiting coagulation, such as TF pathway inhibitor, protein C, or thrombomodulin, raises the possibility of eventual contribution of MPs to an anticoagulant pathway [68,69]. While TF is exposed by EDMPs, TF activity is markedly inhibited by MP-associated tissue factor pathway inhibitor (TFPI). In storage-induced PDMPs, 10% of which contain TF, TF-dependent thrombin generation can only be observed in plasma in which TFPI is neutralized [70]. A balance between TF and TFPI at the MP surface is likely to be a crucial feature in the initiation of blood coagulation, and higher levels of MPs containing TF possibly overcome the TFPI anticoagulant pathway [68]. The effect of activated protein C, which has anticoagulant and anti-inflammatory properties, on endothelial cells and EDMP formation has also been studied [69]. Cultured endothelial cells exposed to activated protein C release EDMP with membrane-bound endothelial protein C receptor. Activated protein C that is bound to this receptor retains its anticoagulant activity in reducing formation of thrombin [69]. Other mechanisms contributing to the regulation of MP procoagulant properties rely on the balance between TNF-α and anti-inflammatory cytokines, such as interleukin (IL)-10. Indeed, endogenous IL-10 was recently reported to downregulate TF expression in monocytes and TF-bound MDMP release, impeding generation of thrombin [71].

### MPs and cellular interactions

**Figure 2:**
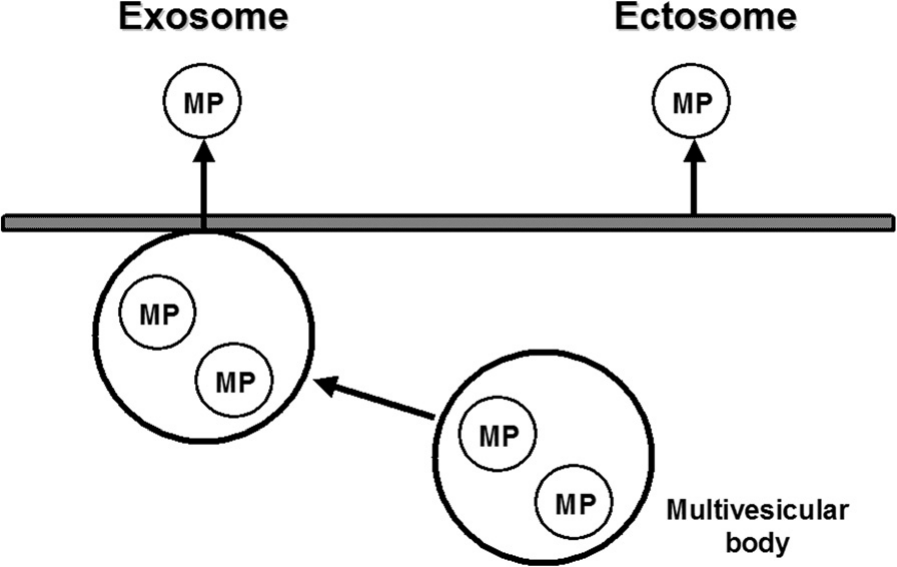
**Different types of secreted membrane microparticles.** Microparticles or pre-microparticles originally exist in multivesicular bodies. Following cellular activation, multivesicular bodies move close to the cellular membrane. Microparticles that are generated in multivesicular bodies are called exosomes once they are secreted. Secreted vesicles can form inside internal compartments from where they are subsequently secreted by fusion of these compartments with the plasma membrane. This microparticle is called an ectosome. Active calpain cleaves the cytoskeleton, leading to the formation of a membrane bleb and ectosome release. Exosome functionates by delivery system of some cellular substances. Ectosome possesses a procoagulant activity.

MPs contain antigens of their cell of origin and can transfer these surface molecules to other cell types and organs [72-74]. These carrier vesicles contain material from the lumen of the donor compartment and expose the cytoplasmic side of this compartment at their outer surface, such as exosomes and ectosomes (Figure 2). The binding of MP surface antigens to their specific counter receptor may activate intracellular signaling pathways. PDMPs show transcellular delivery of unmetabolized arachidonic acid. Additionally, PDMP activation of human vascular endothelial cells and U-937 cells induces *de novo* expression of cyclooxygenase-2 but not cyclooxygenase-1 [30].

The concentrated delivery of PDMP bioactive lipids may modulate multicellular interactions that occur in the early stages of atherogenesis. Nomura et al. [75] also reported that PDMPs that are induced by high shear stress enhance expression of cell adhesion molecules by THP-1 and endothelial cells. PDMPs may contribute to the development of adhesion and participate in vascular damage that is observed in inflammatory disorders. Additionally, PDMPs may possess high mobility and accessibility to leukocytes [76].

### Identification of MPs in the clinical setting

An identification method for MPs is important for clinical studies on MPs. Appropriate sampling conditions, processing, and storage of samples are essential [1]. MPs can be directly quantified in platelet-poor plasma, obtained by serial centrifugation of citrated whole blood. Alternatively, washed MPs can be isolated from platelet-poor plasma by ultracentrifugation before resuspension and analysis.

The most widely used method for studying MPs is flow cytometry because of its simplicity and the wealth of information that can be obtained from the population under study [77]. Platelet-poor plasma or MP suspensions are labeled with fluorescently conjugated monoclonal antibodies. The major advantage of flow cytometry is double staining of MPs to determine the origin/cellular source of MPs. Annexin V binding is used to confirm the phospholipid properties of MPs, although most endothelial MPs do not express this antigen. Antibodies to specific surface antigens expressed on the cells of origin are used to identify the subtype of MP (e.g., anti-GPIb for identification of PDMP). Flow cytometry also allows the criterion of size to be applied to MP analysis, by assessment of the forward light scatter of MPs. The identification of events of a specified size is most accurately performed using calibration beads of a known diameter for comparison [1,26]. Additionally, a variety of cell-specific antibodies have been used, and the specificity that is chosen is likely to influence the results. An example of this situation is that α_IIb_β_3_ and P-selectin are both platelet-specific antigens but α_IIb_β_3_ is present on all platelets, while P-selectin is only found on activated platelets.

The enzyme-linked immunosorbent assay (ELISA) method is an easier and reproducible PDMP assay [78,79]. When using ELISA techniques, PDMPs may be quantitated with reference to a standard curve. This method will hopefully contribute toward the understanding of participation of PDMPs in the clinical setting, if antibodies that are reactive with platelet activation markers, such as P-selectin and soluble CD40 ligand, are used. One of the problems of the ELISA method is the possibility that it contains soluble GPs, such as the GPIb/IX/V complex. Ueba et al. [80-83] measured circulating PDMPs in healthy Japanese individuals using the ELISA method and suggested that PDMPs were positively associated with the level of metabolic syndrome. The use of MP quantification as a clinical tool is still debatable. A large-scale clinical trial for various thrombotic diseases using ELISA kits was performed in Japan [84].

### Atherothrombosis and MPs

Production of PDMPs, EDMPs, and leukocyte-derived MPs can be increased by inflammatory conditions [85,86]. MPs formed by *in vivo* stimulation with a chemotactic peptide in healthy volunteers were able to induce IL-6 and monocyte chemoattractant protein (MCP)-1 release, as well as TF expression, by endothelial cells *in vitro*. The addition of neutrophils to cultured endothelial cells induces the release of IL-6 and IL-8. This effect can be replicated by cell-free supernatant or purified MPs, but not MP-free supernatant [5]. A major feature in atherosclerosis is adhesion of monocytes to endothelial cells, followed by subendothelial transmigration. Cytokines, such as IL-1β and TNF-α, affect this process by inducing synthesis or upregulation of leukocyte-endothelial adhesion molecules. *In vitro* stimulation of monocytes and endothelial cells by high shear stress-induced PDMPs results in significantly increased production of Il-8, IL-1β, and TNF-α [70]. Furthermore, treatment of endothelial cells and monocytes with PDMPs prior to co-incubation modulates monocyte-endothelial cell interactions, by increasing the expression of adhesion molecules on both cell types [70].

Circulating MPs of platelet and leukocytic origins promotes recruitment of inflammatory cells and induces cellular adhesiveness through upregulation of cytokines and cytoadhesions in endothelial cells and monocytes [87]. At high shear stress, PDMP rolling enables delivery of RANTES to inflamed endothelium, thus favoring adhesion of monocytes and infiltration of plaques [32]. Development and progression of atherosclerotic plaques are associated with apoptotic cell death, explaining the presence of a considerable amount of procoagulant MPs within plaques [9]. Furthermore, enhanced apoptosis or activation of leukocytes, SMCs, and endothelium contribute to accumulation of MPs [9,88]. Compared with their circulating counterpart, MPs trapped within the plaque are present at much higher concentrations and display higher thrombogenic potential. In plaques, most of these MPs are from activated leukocytes, a hallmark of inflammation, and from erythrocytes, indicating occurrence of intraplaque hemorrhage, which is a marker of vulnerability of plaques [88]. Atherosclerotic plaques also contain a considerable amount of SMC-derived MPs and EDMPs [9]. Beyond the contribution of MPs to plaque thrombogenicity, MPs can also contribute to instability by mediating the recruitment of inflammatory cells. Therefore, circulating MPs can result in vascular inflammation, endothelial dysfunction, leukocyte adhesion, and recruitment. This could contribute to plaque growth or stent-induced vascular inflammation because MPs convey biological effectors [89].

### Thrombocytopenia

Some anti-platelet antibodies can induce complement-mediated formation of PDMPs and initiate platelet destruction [90,91]. Antiphospholipid antibodies are found in antiphospholipid antibody syndrome (APS). These antibodies are directed against plasma proteins, including β2GPI and prothrombin, which are bound to anionic phospholipids. These phospholipids are abundant on activated platelets, apoptotic cells, and MPs. MP levels are elevated in patients with APS, but not thrombosis, compared with healthy controls [1,92]. Production of procoagulant MPs in APS patients may represent a new pathogenic mechanism for the thrombotic complications of this disease [7,93].

Galli et al. [94] performed a study of PDMPs in thrombotic thrombocytopenic purpura (TTP) and found a rise and fall in PDMP levels with the course of the disease, suggesting that PDMPs are clinically relevant. Jimenez et al. [95] studied the effect of plasma from patients with acute TTP on cultured brain and renal microvascular endothelial cell lines. They found a 5- to 6-fold increase in EDMP generation with TTP plasma compared with controls. Nomura et al. [96] investigated MP levels in patients following allogeneic stem cell transplantation where transplant-related complications included vascular disorders, such as veno-occlusive disease, pulmonary vasculopathy, and thrombotic microangiopathy (TMA). Although only one of the 21 patients who were studied developed TMA/TTP, a continuous rise in platelets, EDMPs, and MDMPs was observed in all of the patients, for up to 4 weeks following transplantation. These findings paralleled an increase in soluble endothelial markers, including vascular cell adhesion molecule (VCAM)-1 and E-selectin.

### Cardiovascular diseases

Procoagulant MPs, and especially EDMP, are elevated in patients with acute coronary syndrome compared with patients with stable anginal symptoms or controls [97]. This finding reflects the degree of acute vascular injury and inflammation at the time of measurement. Steppich et al. [63] reported that in acute myocardial infarction, MPs may also have an anticoagulant function through expression of TFPI and reduction of TF-dependent thrombin generation, which may help limit thrombus formation. Furthermore, EDMP levels are higher in high-risk coronary lesions compared with low-risk lesions [98].

A number of cytokines can induce procoagulant activity in leukocytes [99]. A proinflammatory member of the C-C chemokine family, RANTES, is a potent chemoattractant of memory T lymphocytes, monocytes, eosinophils, and basophils. Several previous studies have suggested that RANTES is an inflammatory mediator in cardiovascular disease [11,85,100]. Additionally, the presence of RANTES may predict restenosis after percutaneous coronary intervention in patients with stable angina [101,102]. PDMPs also relate to the levels of RANTES [11,32,79] and are associated with atherosclerotic events after percutaneous coronary intervention [103].

### Diabetes mellitus

**Figure 3:**
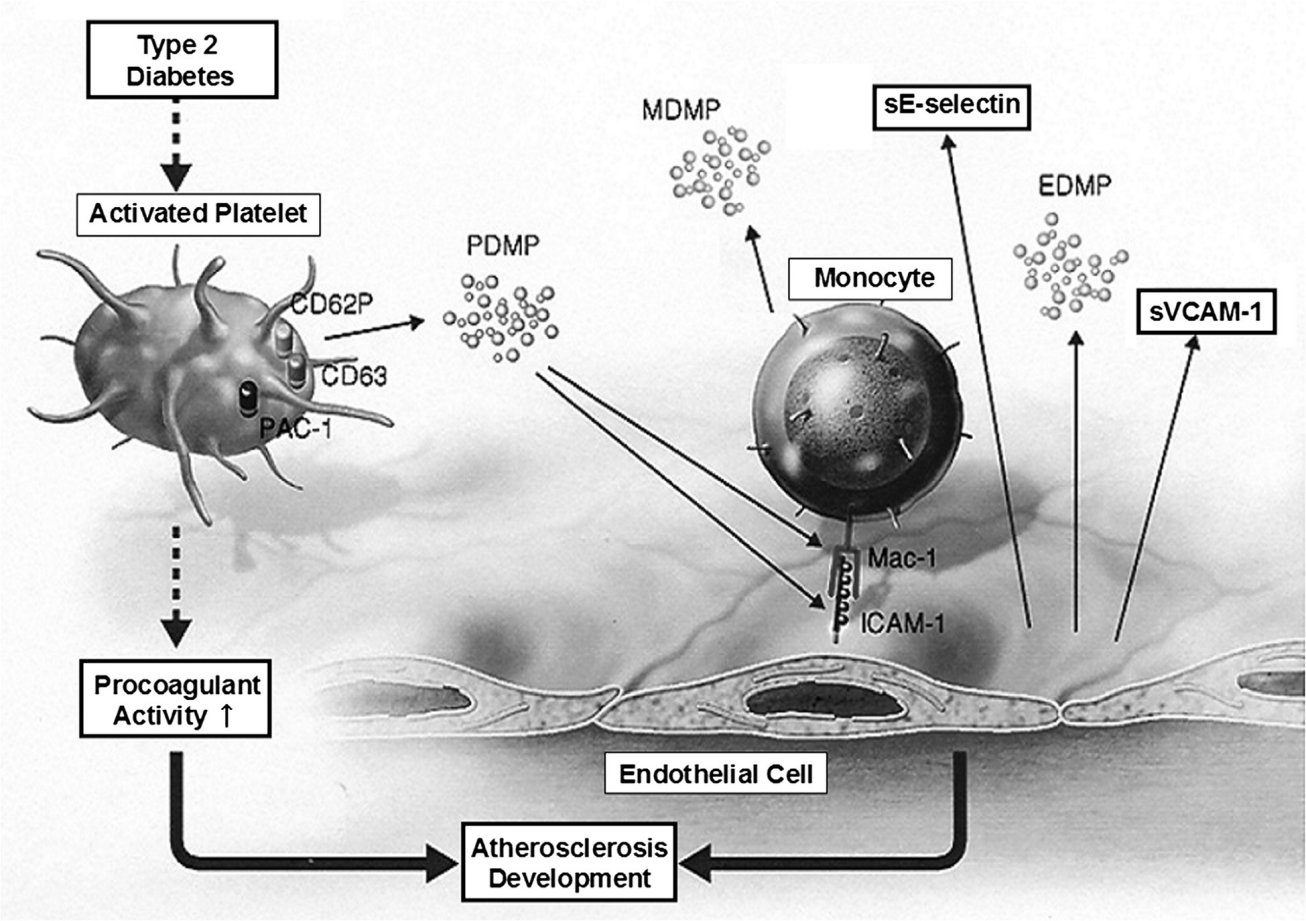
**Role of MPs in type 2 diabetes into atherosclerosis and thrombosis.** Production of PDMPs, MDMPs, and EDMPs can be increased in type 2 diabetes. These MPs contribute to the generation of atherothrombosis in type 2 diabetes. Mac-1: β-2 integrin family in monocyte (CD11b/CD18), ICAM-1: intercellular adhesion molecule-1, VCAM-1: vascular cell adhesion molecule-1.

A few studies on the potential role of PDMPs in diabetic complications have been reported [104-106]. MPs are elevated in diabetic patients. However, studies have found differences in the MP profile in relation to the disease type and the presence or absence of MPs. Sabatier et al. [107] reported that in type 1 diabetes, the procoagulant potential of MPs, as measured by a prothrombinase assay, was elevated and correlated with the degree of glycemic control. In contrast to type 1 diabetes, they found that although total numbers of MPs were elevated in type 2 diabetes, there was no associated increase in their procoagulant potential. Levels of PDMPs and MDMPs are correlated with diabetic complications or the extent of diabetic retinopathy, which is associated with microvascular damage [108-114]. Elevated EDMP levels are predictive for the presence of coronary artery lesions, and they are a more significant independent risk factor than the length of diabetic disease, lipid levels, or presence of hypertension [115]. Interestingly, elevated EDMP levels are predictive in identifying a subpopulation of diabetic patients without typical anginal symptoms who have angiographic evidence of coronary artery disease. Production of PDMP, MDMP, and EDMP can be increased in type 2 diabetes. These MPs contribute to the generation of atherothrombosis in type 2 diabetes (Figure 3).

### Sepsis and disseminated intravascular coagulation

Disseminated intravascular coagulation (DIC) is frequently complicated by various diseases [116]. Coagulation abnormalities and thrombocytopenia are common in DIC, and the extent of hemostatic disorders appears to correlate with disease severity. In particular, septic shock-induced DIC contributes to multiple organ failure. In DIC patients, thrombin generation may react with thrombin receptors located on platelets and results in the generation of PDMPs by activation of platelets. Additionally, HMGB1 also plays a role in the pathogenesis of DIC because plasma HMGB1 levels correlate with the DIC score [117]. Nomura et al. [118] described the role of PDMPs and HMGB1 in DIC patients with hematological malignancies.

**Figure 4:**
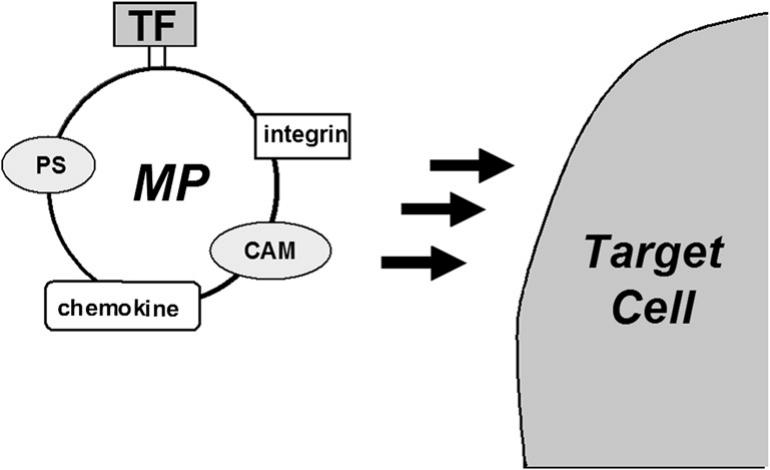
**Role of TF on MPs in activation of target cells.** MPs can carry some substances, such as integrin, cell adhesion molecule, chemokines, phospholipids, and TF. TF mainly contributes to activation of the extrinsic coagulation system. PS, phosphatidylserine; CAM, cell adhesion molecule.

Activation of leukocytes and endothelial cells are also observed in DIC. These contribute to the generation of EDMPs, MDMPs, and others. Delabranche et al. [119] reported that EDMPs are relevant biomarkers of septic shock-induced DIC and can be used to evaluate early vascular injury. Furthermore, Hellum et al. [120] recently reported that MPs that were obtained from patients with meningococcal septic shock displayed more efficient TF-dependent thrombin generation and clot formation compared with MPs from meningitis patients. They concluded that MP-associated TF activity was closely associated with plasma lipopolysaccharide levels in the septic shock group. These changes are thought to be dependent on TF on MPs (Figure 4).

### Other clinical situations

Elevated PDMP levels have been detected in other clinical conditions, including infectious diseases [121,122], peripheral blood stem cell harvest [123-125], progressive systemic sclerosis [126], and arteriosclerosis obliterans [127,128]. Additionally, PDMPs are used for monitoring of anti-thrombotic therapy [110,129].

PDMPs bind to hematopoietic cells, resulting in enhancement of their engraftment by stimulating proliferation, survival, adhesion, and chemotaxis [130-132]. Majka et al. [133] also reported that PDMPs transfer platelet-specific immunoreactive antigens to the surface of endothelial and hematopoietic cells. Another study showed that endothelial progenitor cell-derived MPs were incorporated in endothelial cells by interaction with α4 and β1 integrins expressed on the MP surface [134]. This finding suggested that endothelial progenitor cells may activate angiogenesis in endothelial cells by releasing MPs able to trigger an angiogenic program. CD42b-negative/α4-integrin-positive MPs show the same changes as stroma cell-derived factor 1 and soluble CD40 ligand, with an increase in CD34^+^ stem cells during peripheral blood stem cell harvest [118]. Janowska-Wieczorek et al. [135] suggested that MPs that are actively released from cells may play an important role in cell-to-cell communication. Results from other studies have also supported this view [124,134,136]. Furthermore, elevation of EDMPs in patients following allogeneic stem cell transplantation may be associated with some transplant-related complications, such as graft-versus-host-disease (GVHD) and TMA [96,125,131].

In lung cancer patients, PDMPs induce metastasis and angiogenesis, [135] and MDMPs may be a sign of vascular complications [137]. In patients with various types of cancer, PDMPs possess CXCR4 and contribute to chemotaxis by stromal cell-derived factor 1, resulting in progression or metastasis of cancer [138,139]. TF overexpression by cancer cells is closely associated with tumor progression, and TF-expressing MPs that are shed by cancer cells are linked to the genetic status of cancer [140-144].

A current feature of clinical applications regarding MPs is detection of TF-expressing MPs that are generated by apoptosis. Fas ligand- or TNF-related apoptosis-inducing ligand-positive MPs have been isolated from sera of patients with cancer, and these MPs can induce T-cell apoptosis [145-147]. The pattern of procoagulant MPs that are released during acute allograft rejection suggests endothelial cell activation and Fas-mediated apoptosis [148]. Procoagulant MPs in pulmonary arterial hypertension also belong to apoptotic EDMPs [149].

## Conclusion

We have summarized the literature to date that is relevant to MPs, including a growing list of clinical disorders that are associated with elevated MP levels. MPs were initially thought to be small particles with procoagulant activity. However, the possibility that MPs evoke cellular responses in the immediate microenvironments where they are formed is now under investigation.

## Abbreviations

APS: antiphospholipid antibody syndrome
CXCR4: CXC receptor 4, CD184
DAMPs: damage-associated molecular patterns
DIC: disseminated intravascular coagulation
EDMP: endothelial cell-derived microparticle
ELISA: enzyme-linked immunosorbent assay
FVII: factor VII
GP: glycoprotein
GVHD: graft-versus-host disease
HMGB1: high-mobility group-B1 DNA-binding protein 1
IL-6: interleukin-6
IL-10: interleukin-10
MCP-1: monocyte chemoattractant protein-1
MDMP: monocyte-derived microparticle
MP: microparticle
PDMP: platelet-derived microparticle
PIP_2_: phosphatidyl inositol phosphate 2
PS: phosphatidylserine
RANTES: regulated on activation, normal T-cell expressed and secreted
SMC: smooth muscle cell
TF: tissue factor
TFPI: tissue factor pathway inhibitor
TMA: thrombotic microangiopathy
TNF-α: tumor necrosis factor-α
TTP: thrombotic thrombocytopenic purpura
VCAM-1: vascular cell adhesion molecule-1
